# Formula for the Cross-Sectional Area of the Muscles of the Third Lumbar Vertebra Level from the Twelfth Thoracic Vertebra Level Slice on Computed Tomography

**DOI:** 10.3390/geriatrics5030047

**Published:** 2020-09-05

**Authors:** Yuria Ishida, Keisuke Maeda, Yosuke Yamanaka, Remi Matsuyama, Ryoko Kato, Makoto Yamaguchi, Tomoyuki Nonogaki, Akio Shimizu, Junko Ueshima, Kenta Murotani, Naoharu Mori

**Affiliations:** 1 Department of Nutrition, Aichi Medical University Hospital, 1-1 Yazakokarimata, Nagakute, Aichi 480-1195, Japan; okuda.yuria.785@mail.aichi-med-u.ac.jp; 2 Department of Geriatric Medicine, National Center for Geriatrics and Gerontology, 7-430 Morioka, Obu, Aichi 474-8511, Japan; 3 Department of Palliative and Supportive Medicine, Graduate School of Medicine, Aichi Medical University, 1-1 Yazakokarimata, Nagakute, Aichi 480-1195, Japan; nmori@aichi-med-u.ac.jp; 4 Department of Oral and Maxillofacial Surgery, Graduate School of Medicine, Aichi Medical University, 1-1 Yazakokarimata, Nagakute, Aichi 480-1195, Japan; yamanaka.yousuke.694@mail.aichi-med-u.ac.jp (Y.Y.); matsuyama.remi.721@mail.aichi-med-u.ac.jp (R.M.); 5 Department of Pharmacy, Aichi Medical University Hospital, 1-1 Yazakokarimata, Nagakute, Aichi 480-1195, Japan; inuduka.ryouko.907@mail.aichi-med-u.ac.jp (R.K.); nonogaki.tomoyuki.562@mail.aichi-med-u.ac.jp (T.N.); 6 Department of Nephrology and Rheumatology, Aichi Medical University, 1-1 Yazakokarimata, Nagakute, Aichi 480-1195, Japan; yamaguchi.makoto.231@mail.aichi-med-u.ac.jp; 7 Department of Nutrition, Hamamatsu City Rehabilitation Hospital, 1-6-1 Wagokita, Naka-Ku, Hamamatsu, Shizuoka 433-8511, Japan; a.shimizu.diet@gmail.com; 8 Department of Clinical Nutrition and Food Service, NTT Medical Center Tokyo, 5-9-22 Higashi-Gotanda, Shinagawa-Ku, Tokyo 141-0022, Japan; j.ueshima@gmail.com; 9 Biostatistics Center, Kurume University, 67 Asahimachi, Kurume, Fukuoka 830-0011, Japan; kmurotani@med.kurume-u.ac.jp; 10 Nutritional Therapy Support Center, Aichi Medical University Hospital, 1-1 Yazakokarimata, Nagakute, Aichi 480-1195, Japan

**Keywords:** muscles, computed tomography, equation

## Abstract

The purpose of this study was to investigate a means by which to reflect muscle mass using chest computed tomography (CT). A cross-sectional study was conducted with patients aged ≥ 65 years having abdominal and chest CT scans. The formula to predict third lumbar vertebra (L3) cross-sectional area (CSA) of the muscles from the erector muscles of the spine at the twelfth thoracic vertebra (Th12) level slice on CT was created using the five-fold cross-validation method. Correlation between predicted L3 CSA and measured L3 CSA of the muscles was assessed by intraclass correlation coefficients (ICC) and correlation coefficients (*r*) in the data of the development, and predictability was examined with accuracy and *F*-values in the validation study. The development study included 161 patients. The developed formula was as follows: −1006.38 + 16.29 × age + 1161.80 × sex (if female, 0; if male, 1) + 55.91 × body weight + 2.22 × CSA of the erector muscles at Th12. The formula demonstrated strong concordance and correlation (ICC = 0.849 [0.800–0.887] and *r* = 0.858 [0.811–0.894]). The validation study included 34 patients. The accuracy and *F*-value between predicted CSA and measured CSA were high (accuracy = 0.889–0.944, *F*-value = 0.931–0.968). We developed a formula predicting CSA at L3 using Th12 CT slice. This formula could be used to assess decreased muscle mass even with chest CT alone.

## 1. Introduction

Globally, sarcopenia has received a great deal of attention as an issue in the field of geriatric nutrition [1]. Sarcopenia, caused by age, disease, and other factors, is associated with increased adverse outcomes, such as high fall rates, high fracture rates, and increased mortality [2,3]. Expert groups such as the European Working Group on Sarcopenia in Older People (EWGSOP) and the Asian Working Group for Sarcopenia (AWGS) have defined sarcopenia as decreased muscle mass and decline of muscle strength [1,2]. EWGSOP suggests that magnetic resonance imaging, computed tomography (CT), dual-energy X-ray absorptiometry, and bioelectrical impedance analysis are appropriate methods for measuring skeletal muscle mass to determine decreased muscle mass [2]. Moreover, according to the EWGSOP, CT assessment of decreased skeletal muscle mass is the gold standard [2]. In general, CT scans are frequently used in clinical practice because they can detect lesions of various diseases by taking cross-sectional images of the body using X-rays. Therefore, CT images are an essential source of information for assessing skeletal muscle mass and the process of diagnosing sarcopenia.

The cross-sectional area (CSA) of muscle measured on CT images strongly correlates with whole-body skeletal muscle mass [4]. For example, the CSA of muscles as assessed by CT images of the abdomen and thighs is strongly correlated with skeletal muscle mass of the whole body among both sexes [5]. In particular, the skeletal muscle index (SMI), a height squared-adjusted CSA of the muscles at the third lumbar vertebra (L3) on CT has a number of cutoff values referring to decreased skeletal muscle mass [6,7,8,9]. In addition, SMI is associated with negative clinical outcomes, including prolonged hospitalization and increased mortality [10]. One important reason for the extensive use of the L3 level is its inclusion in typical abdominal CTs. Therefore, the CSA of the muscles at L3 measured on CT has been used generically in the assessment of skeletal muscle mass [10]. However, L3 is not always included in the typical chest CT images. For example, lung cancer, pneumonia, thoracic aortic aneurysm, and heart disease are the most common diseases that affect the older population, and chest CT is used to diagnose and differentiate between these diseases. However, among older patients, the cutoff value detecting low skeletal muscle mass in CSA of the muscles measured on chest CT has not been clarified. Therefore, patients for whom abdominal CT images were not obtained cannot be evaluated for low skeletal muscle mass. If the evaluation of skeletal muscle mass can be performed using chest CT, numerous patients could be evaluated for sarcopenia in clinical practice. Therefore, the purpose of this study was to investigate validated methods for assessing skeletal muscle mass using chest, rather than abdominal, CT.

## 2. Materials and Methods

### 2.1. Study Design and Participants

This cross-sectional study employed existing medical records to evaluate the eligibility of patients aged ≥65 years who were admitted to a 900-bed university hospital and underwent abdominal and chest CT on the same day. The study included two periods, namely the development and validation periods. The development study was conducted to create a formula to predict L3 level CSA of the muscles, using Th12 level of the erector muscles of the spine on CT. The validation study was conducted to evaluate the accuracy of the prediction formulas. The development study included patients from October 2019 to March 2020, and the validation study included patients from April to May 2020. Exclusion criteria were patients whose date of CT scan was performed more than 15 days after admission, and/or if the CT image was defined as difficult to analyze due to poor quality. The ethics committee of the hospital approved this study (ID: 2020-043), which was conducted in compliance with the principles of the Declaration of Helsinki. The need for informed consent was waived by the ethics committee, given the retrospective nature of the study. Instead, an opt-out procedure through an online announcement on the hospital webpage was used, to provide an opportunity for patients to withdraw or to tacitly consent to participate in the study.

### 2.2. Variables

Patient data included age, sex, body weight, body height, and nutritional indices were collected. Nutritional indices included body mass index (BMI) and the Mini Nutritional Assessment-Short Form (MNA-SF) [11]. BMI was calculated by the body weight [kg] divided by the square of the body height [m]. MNA-SF was assessed by trained nurses upon admission and scored on a total range of 0–14 points. Lower scores indicate poor nutritional status according to the MNA-SF. The primary disease related to hospitalization was reviewed based on the medical chart record from the attending physician, and the disease category was classified according to the International Classification of Disease-10 (ICD) -10) codes.

### 2.3. Measurement of CSA of the Muscles

**Figure 1:**
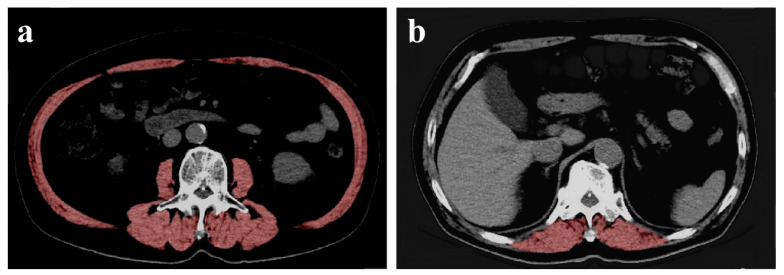
Example of L3 and Th12 delineation using ImageJ. (**a**,**b**) show the cross-sectional computed tomography (CT) images of L3 and Th12, respectively. The cross-sectional area of the muscle in the red area is measured.

CT slices at L3 and Th12 levels were identified from a series of non-enhanced CT images for each patient. Th12-level slices were selected because Th12 did not typically include the peri-scapular muscles. The conditions for abdominal CT imaging at the hospital were 120 KV, 240 mA, helical rotation time of 0.5 s, slice thickness of 2.0–5.0 mm, and 0.8 pitch (SIEMENS SOMATOM Definition AS+). The slices with the clearest vertebral arch were determined to be retained for analyses. Image analysis for measuring CSA of the muscles was performed using ImageJ software [12]. Digital Imaging and Communications in Medicine format images were imported into ImageJ software and analyzed. The muscle boundaries were traced manually under the Hounsfield Unit thresholds of −25 to 150 [13]. Examples of measurement of CSA of the muscles at L3 and Th12 level using ImageJ are shown in Figure 1. The CSA in the red area in Figure 1 was analyzed. The muscles included in the analyses were in the psoas major muscle, erector spinae muscle, quadratus lumborum, transverse abdominis, external oblique, internal oblique muscles, and rectus abdominis at L3, and only the erector spinae muscles at Th12. The CT images to be analyzed were randomly assigned to six trained researchers.

### 2.4. Skeletal Muscle Mass Index

SMI was calculated by the CSA [cm^2^] of the skeletal muscles extracted from the CT images divided by the square of the patient’s height [m] [14]. Low SMI was determined using following previously reported cut-off values: (1) Carey’s criteria [6], <39 cm^2^/m^2^ for females and <50 cm^2^/m^2^ for males; (2) Montano’s criteria [7], <42 cm^2^/m^2^ for females and <50 cm^2^/m^2^ for males; (3) the European Society of Clinical Nutrition and Metabolism (ESPEN) criteria [8], <39 cm^2^/m^2^ for females and <55 cm^2^/m^2^ for males; and (4) Prado’s criteria [9], <38.5 cm^2^/m^2^ for females and <52.4 cm^2^/m^2^ for males.

### 2.5. Statistical Analyses

Quantitative variables are presented as mean ± standard deviation for parametric variables, based on the histogram. Nonparametric variables were presented as median [interquartile range]. Categorical data are expressed as a percentage of frequency. The five-fold cross validation method [15] was used to create the prediction formula for CSA of the muscles at L3 level from that of CSA of the muscles at Th12. First, the data set was randomly divided into five groups, and a preliminary prediction formula was created using the four of the five groups of the data sets. Next, we tested the performance of the preliminary prediction formula on the single remaining group. The procedures for creating a preliminary prediction formula and testing its performance were repeated for every 4-to−1 group combination. The coefficients in the final prediction formula were averaged in each variable of the formulas. According to the study by Swartz et al., the variables used to create the prediction formula included age, sex, weight, and CSA of the muscles at Th12 level [13]. In order to assess how well the predicted CSA of the muscles at L3 correlated with the measured CSA of the muscles at L3 level, the correlation coefficient (*r*) and the intraclass correlation coefficients (ICC) were examined. For the correlation coefficient (*r*), 0 indicates no correlation and the correlation is stronger as the coefficient approaches an absolute value of 1 [16]. ICC values less than 0.5 are indicative of poor, values between 0.5 and 0.75 indicate moderate, values between 0.75 and 0.9 indicate good and values greater than 0.90 indicate excellent reliability [17]. In addition, sensitivity, specificity, positive predictive value (PPV), negative predictive value (NPV), accuracy, and *F*-values adapting known cut-off values discriminating decreased muscle mass between the predicted SMI based on predicted CSA of the muscles and the measured SMI based on measured CSA of the muscles were examined. All statistical operations were performed using SPSS ver.24 (IBM Japan, Tokyo, Japan). A *p*-value <0.05 was considered statistically significant.

## 3. Results

In the development study, 307 patients who underwent abdominal and chest CT scans were considered for eligibility. One hundred forty-three patients whose CT scans were performed more than 15 days after hospitalization and three patients whose CT images were of poor quality were excluded. Finally, 161 patients were analyzed in the development study. The mean age was 76.9 ± 6.5 years (Table 1). The dominant reasons for hospitalization were digestive disease (44.1%), neoplasms (24.8%), and circulatory system disease (15.5%) classified by ICD-10 code (Table 1).

**Figure 2:**
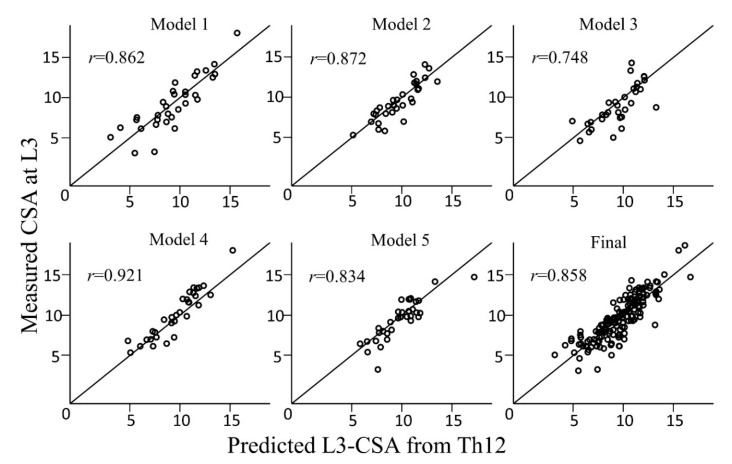
Scatter plot of measured cross-sectional area (CSA) of the muscles and predicted CSA of the muscles in each preliminary model and the final model.

The final prediction formula derived from the five-fold cross validation was as follows: −1006.38 + 16.29 × age + 1161.80 × sex (if female, 0; if male, 1) + 55.91 × body weight + 2.22 × CSA of the muscles at Th12 (Table 2). The *r* and ICC of the final predictive formula were 0.858 [0.811–0.894] and 0.849 [0.800–0.887], respectively (Figure 2).

There was a significant correlation between the cross-sectional area (CSA) of the skeletal muscle mass at the L3 level and the muscles at the Th12 level (*r* = 0.858). The Y- and X-axes indicate the measured CSA of the muscle mass and predicted CSA of the muscle mass, respectively.

The prevalence of low SMI in terms of predicted SMI was 91.9–96.3%. The low SMI according to the known cut-off values between the predicted and measured SMI showed high accuracy (accuracy = 0.913–0.944) and a high prediction rate (*F*-value = 0.951–0.970) (Table 3). The accuracy of Carey et al.’s criteria for low SMI according to disease that led to hospitalization was 0.944, 0.850, and 0.960 for digestive disease, neoplasms, and circulatory system disease, respectively.

In the validation study, 57 participants were assessed for eligibility. Twenty-two patients whose CT scans were conducted more than 15 days after hospitalization, and one patient whose CT image was poor quality were excluded, and finally, 34 patients were analyzed. The mean age was 75.3 ± 5.9 years. The ICC between the predicted CSA and the measured CSA indicated good (0.846 [0.720–0.910]). The prevalence of low SMI in terms of predicted SMI was 83.3–86.1%. The low SMI according to the known cut-off values between the predicted and measured SMI showed high accuracy (accuracy = 0.889–0.944) and a high prediction rate (*F*-value = 0.931–0.968) (Table 3).

## 4. Discussion

We investigated a predictive equation for the diagnosis of decreased skeletal muscle mass using chest CT. We observed the following two novel points in this study. First, we have developed a prediction formula to convert CT slice data from erector muscles of the spine at Th12 level to CSA of the muscles at L3 level. Second, the accuracy of the developed formula to discriminate decreased skeletal muscle mass was applicable. CSA measurement of the muscles on chest CT images may facilitate assessment of decreased skeletal muscle mass and aid in the detection of sarcopenia without the need of abdominal CT.

The prediction formula for CSA of the muscles at L3 level using CSA of the muscles at Th12 level on CT is −1006.38 + 16.29 × age + 1161.80 × sex (if female, 0; if male, 1) + 55.91 × body weight + 2.22 × CSA of the muscles at Th12. Several formulas for CSA of the muscles at L3 level from other level slices have been reported [13,18]. Swartz et al. developed an estimation formula to convert CSA of the muscles at the third cervical level to L3 level CSA of the muscles [13]. In the current study, the items in the predictive formula for converting Th12 level CSA to L3 level CSA of the muscles were age, body weight, sex, and CSA of the muscles, as in previous reports. In this study, we did not include height in the development of the prediction equation given that body weight and height are often proportional to each other. The coefficients of each variable in the prediction formula developed in the current study were positive for all items. In other words, we demonstrated that males and heavier weight have a larger predicted L3 level CSA of muscles. CSA of muscle mass is known to be larger among individuals with high BMI and larger among males [19]. The study by Swartz et al. also revealed the items for body weight and sex in the prediction formula to be positive [13], consistent with our findings. In addition, the CSA of muscle mass decreases with age [19], but the CSA of muscle mass at L3 displays a smaller decrease than the CSA of muscle mass at Th12 [20]. Therefore, the coefficients for the age of the developed prediction formula for converting Th12 to L3 are considered to be positive. Moreover, the *r* and ICC were 0.858 and 0.849, respectively. An ICC of 0.75 to 0.9 indicates good reliability [17]. In this study, strong correlations were observed, which demonstrates the developed construct validity.

The accuracy of the low SMI determined by the predicted SMI was high. Several criteria for determining decreased skeletal muscle mass on SMI based on CT have previously been reported [6,7,8,9]. Carey et al. used survival as an external criterion, while Montano et al. and Prado et al. used mortality as an external criterion for the cutoff values of SMI on L3 level CT slices [6,7,8]. The ESPEN criteria were based on the decreased appendicular skeletal muscle mass index as an external criterion used in the diagnosis of sarcopenia as proposed by the EWGSOP [9]. In the current study, we adapted the cutoff values of the four criteria and evaluated the accuracy of each. Regardless of the differences of reported cutoff values, the accuracy of the low SMI determined by the predicted SMI demonstrated high accuracy (accuracy = 0.913–0.944) and a high prediction rate (*F*-value = 0.951–0.970) in the population of the development study. Additionally, the accuracy according to disease was high (accuracy = 0.850–0.960). Further, the current study included an accuracy analysis adapted to the population from a different time in the validation study to test the predictive validity. The results were considered consistent with those in the development study (accuracy = 0.889–0.944, *F*-value = 0.931–0.968). Given the accuracy of the previously reported cutoff values for decreased skeletal muscle mass and the results of the current study, the predictive formula developed in this study is appropriate for the diagnosis of decreased skeletal muscle mass.

There are several limitations to this study. First, the measurement of CSA of the muscles included one person per slice. Therefore, the values of each measured CSA of the muscles may have been biased. However, the images to be analyzed were randomly assigned and measured to reduce the bias. Secondly, this study was conducted at a hospital in Japan. Therefore, there may be issues with generalization to a different racial population.

## 5. Conclusions

The study developed a formula for assessing skeletal muscle mass and diagnosing decreased skeletal muscle mass based on chest CT. The formula would allow physicians more opportunities to assess and diagnose sarcopenia in clinical practice. It is necessary to verify whether sarcopenia assessed using the predictive formula is associated with clinical outcomes.

## Figures and Tables

**Table 1:** Characteristics of patients in the development study.

	Overall (*n* = 161)
Age, year	76.9 ± 6.5
Sex, male (%)	105 (65.2)
Female (%)	56 (34.8)
Weight, kg	57.0 ± 11.6
Height, cm	159.8 ± 9.0
Body mass index, kg/m^2^	22.1 ± 3.7
CSA of the muscles mass at L3, cm^2^	94.4 ± 27.3
CSA of the muscles mass at Th12, cm^2^	24.3 ± 6.9
SMI, cm^2^/m^2^	37.4 ± 8.7
MNA-SF, score	12 [10–13]
Disease related to hospitalization, *n* (%)	
Digestive disease	71 (44.1)
Neoplasms	40 (24.8)
Circulatory system disease	25 (15.5)
Injury	6 (3.7)
Genitourinary system disease	5 (3.1)
Others	14 (8.7)

Abbreviations: CSA, cross-sectional area; SMI, skeletal muscle index; MNA-SF, Mini Nutritional Assessment Short Form.

**Table 2:** Several prediction models in a five-fold cross validation.

Preliminary Models	Intercept	Age	Sex (male = 1, female = 0)	Weight	CSA of the Muscles at Th12	Spearman’s Rank Correlation Coefficient (*r*)	ICC
1	−1443.30	21.16	948.92	50.60	2.43	0.862 [0.733–0.931]	0.859 [0.732–0.928]
2	58.78	5.68	1352.35	55.31	2.10	0.872 [0.752–0.936]	0.863 [0.740–0.931]
3	−1139.18	20.93	1284.43	48.98	2.29	0.748 [0.540–0.870]	0.724 [0.509–0.855]
4	−1707.10	20.64	1073.13	65.51	2.12	0.921 [0.844–0.961]	0.897 [0.802–0.948]
5	−801.11	13.05	1150.18	59.13	2.16	0.834 [0.688–0.915]	0.823 [0.673–0.908]
Average	−1006.38	16.29	1161.80	55.91	2.22	0.858 [0.811–0.894]	0.849 [0.800–0.887]

Abbreviations: CSA, cross-sectional area; ICC, intraclass correlation coefficients.

**Table 3:** Accuracy of the predicted SMI using several external criteria.

Reported Cutoff	Examined Period	Sensitivity/	Positive Predictive Value/	Accuracy	*F*-Value
		Specificity	Negative Predictive Value		
Carey et al.	Development	0.979 [0.939–0.996]/	0.926 [0.871–0.962]/	0.913 [0.858–0.952]	0.951
		0.476 [0.257–0.702]	0.769 [0.462–0.950]		
	Validation	0.964 [0.817–0.999]	0.900 [0.735–0.979]	0.889 [0.739–0.969]	0.931
		0.625 [0.245–0.915]	0.833 [0.359–0.996]		
Montano et al.	Development	0.980 [0.942–0.996]/	0.960 [0.915–0.985]/	0.944 [0.897–0.974]	0.970
		0.571 [0.289–0.823]	0.727 [0.390–0.940]		
	Validation	0.938 [0.792–0.992]	1.000 [0.833–1.000]	0.944 [0.813–0.993]	0.968
		1.000 [0.284–1.000]	0.667 [0.223–0.957]		
ESPEN	Development	0.986 [0.952–0.998]/	0.935 [0.885–0.969]/	0.925 [0.873–0.961]	0.960
		0.286 [0.084–0.581]	0.667 [0.223-0.957]		
	Validation	0.966 [0.822–0.999]	0.903 [0.742-0.980]	0.889 [0.739–0.969]	0.933
		0.571 [0.184–0.901]	0.800 [0.284–0.995]		
Prado et al.	Development	0.986 [0.951–0.998]/	0.928 [0.875–0.964]/	0.919 [0.866–0.956]	0.956
		0.353 [0.142–0.617]	0.750 [0.349–0.968]		
	Validation	0.966 [0.822–0.999]	0.903 [0.742–0.980]	0.889 [0.739–0.969]	0.933
		0.571 [0.184–0.901]	0.800 [0.284–0.995]		

In development study for SMI calculated by the prediction formula, the prevalence of low SMI was 148 (91.9%) for the Carey et al. criteria, 150 (93.2%) for the Montano et al. criteria, 155 (96.3%) for the ESPEN criteria, and 153 (95.0%) for the Prado et al. criteria. In validation study for SMI calculated by the prediction formula, the prevalence of low SMI was 30 (83.3%) for the Carey et al. criteria, 30 (83.3%) for the Montano et al. criteria, 31 (86.1%) for the ESPEN criteria, and 31 (86.1%) for the Prado et al. criteria. Abbreviations: SMI, skeletal muscle mass index; MCC, Matthews Correlation Coefficient; ESPEN, European Society of Clinical Nutrition and Metabolism.
