# Addendum: Ishida et al. Formula for the Cross-Sectional Area of the Muscles of the Third Lumbar Vertebra Level from the Twelfth Thoracic Vertebra Level Slice on Computed Tomography. *Geriatrics* 2020, *5*, 47

**DOI:** 10.3390/geriatrics6020056

**Published:** 2021-06-02

**Authors:** Yuria Ishida, Keisuke Maeda, Yosuke Yamanaka, Remi Matsuyama, Ryoko Kato, Makoto Yamaguchi, Tomoyuki Nonogaki, Akio Shimizu, Junko Ueshima, Kenta Murotani, Naoharu Mori

**Affiliations:** 1 Department of Nutrition, Aichi Medical University Hospital, 1-1 Yazakokarimata, Nagakute, Aichi 480-1195, Japan; okuda.yuria.785@mail.aichi-med-u.ac.jp; 2 Department of Geriatric Medicine, National Center for Geriatrics and Gerontology, 7-430 Morioka, Obu, Aichi 474-8511, Japan; 3 Department of Palliative and Supportive Medicine, Graduate School of Medicine, Aichi Medical University, 1-1 Yazakokarimata, Nagakute, Aichi 480-1195, Japan; a.shimizu.diet@gmail.com (A.S.); j.ueshima@gmail.com (J.U.); nmori@aichi-med-u.ac.jp (N.M.); 4 Department of Oral and Maxillofacial Surgery, Graduate School of Medicine, Aichi Medical University, 1-1 Yazakokarimata, Nagakute, Aichi 480-1195, Japan; yamanaka.yousuke.694@mail.aichi-med-u.ac.jp (Y.Y.); matsuyama.remi.721@mail.aichi-med-u.ac.jp (R.M.); 5 Department of Pharmacy, Aichi Medical University Hospital, 1-1 Yazakokarimata, Nagakute, Aichi 480-1195, Japan; inuduka.ryouko.907@mail.aichi-med-u.ac.jp (R.K.); nonogaki.tomoyuki.562@mail.aichi-med-u.ac.jp (T.N.); 6 Department of Nephrology and Rheumatology, Aichi Medical University, 1-1 Yazakokarimata, Nagakute, Aichi 480-1195, Japan; yamaguchi.makoto.231@mail.aichi-med-u.ac.jp; 7 Department of Nutrition, Hamamatsu City Rehabilitation Hospital, 1-6-1 Wagokita, Naka-Ku, Hamamatsu, Shizuoka 433-8511, Japan; 8 Department of Clinical Nutrition and Food Service, NTT Medical Center Tokyo, 5-9-22 Higashi-Gotanda, Shinagawa-Ku, Tokyo 141-0022, Japan; 9 Biostatistics Center, Kurume University, 67 Asahimachi, Kurume, Fukuoka 830-0011, Japan; kmurotani@med.kurume-u.ac.jp; 10 Nutritional Therapy Support Center, Aichi Medical University Hospital, 1-1 Yazakokarimata, Nagakute, Aichi 480-1195, Japan

The authors would like to make an addendum to their published paper [1]. 

In the published paper, the units in the developed formula in the study were not described. This may confuse followers regarding how to use the formula. Therefore, we would sincerely like to add the units into the formula. The formula described in the published version is: “−1006.38 + 16.29 × age + 1161.80 × sex (if female, 0; if male, 1) + 55.91 × body weight + 2.22 × CSA of the erector muscles at Th12” and can be found in the Abstract and the Methods section. We would like to make the following amendment: “−1006.38 + 16.29 × age [years] + 1161.80 × sex (if female, 0; if male, 1) + 55.91 × body weight [kg] + 2.22 × CSA of the erector muscles at Th12 [mm^2^]”.

The change does not affect the scientific results.

The rest of the manuscript does not need to be changed. The authors would like to apologize for any inconvenience caused.
